# Comparing the Curative Effects between Femtosecond Laser-Assisted Cataract Surgery and Conventional Phacoemulsification Surgery: A Meta-Analysis

**DOI:** 10.1371/journal.pone.0152088

**Published:** 2016-03-21

**Authors:** Xinyi Chen, Kailin Chen, Jiliang He, Ke Yao

**Affiliations:** 1 Eye Center, Second Affiliated Hospital of School of Medicine, Zhejiang University, Hangzhou, Zhejiang, China; 2 Institutes of Environmental Medicine, School of Medicine, Zhejiang University, Zhejiang, China; National Eye Institute, UNITED STATES

## Abstract

**Purpose:**

To compare the outcomes of femtosecond laser-assisted cataract surgery (FLACS) with those of conventional phacoemulsification surgery (CPS) for age-related cataracts.

**Methods:**

A comprehensive literature search of PubMed, EMBASE, and the Cochrane Controlled Trials Register was conducted to identify randomized controlled trials (RCT) and comparative cohort studies comparing FLACS with CPS. Endothelial cell loss percentage (ECL%), central corneal thickness (CCT), corrected and uncorrected distant visual acuity (CDVA and UDVA), and mean absolute error (MAE) of refraction were used as primary outcomes. Secondary outcomes included surgically induced astigmatism (SIA), mean effective phacoemulsification time (EPT), phacoemulsification power and circularity of the capsulorhexis.

**Results:**

Nine RCTs and fifteen cohort studies including 4,903 eyes (2,861 in the FLACS group and 2,072 in the CPS group) were identified. There were significant differences between the two groups in ECL% at one week, about one month and three months postoperatively, in CCT at one day, about one month postoperatively and at the final follow-up, in CDVA at one week postoperatively, and in UDVA at the final follow-up. Significant differences were also observed in MAE, EPT, phacoemulsification power, and the circularity of capsulorhexis. However, no significant differences were observed in CDVA at one week postoperatively or in surgically induced astigmatism.

**Conclusions:**

Compared to CPS, FLACS is a safer and more effective method for reducing endothelial cell loss and postoperative central corneal thickening as well as achieving better and faster visual rehabilitation and refractive outcomes. However, there is no difference in final CDVA and surgically induced astigmatism between the two groups.

## Introduction

Cataract is the leading cause of reversible blindness worldwide, and it can be effectively treated with cataract surgery. With the development of improved equipment and technology over the past few years, cataract surgery is now one of the safest and most successful major surgical procedures performed worldwide. [1] Phacoemulsification is currently the predominant surgical technique employed in developed countries. [2,3] Although conventional phacoemulsification surgery (CPS) provides good visual acuity and rarely causes complications, patients still expect to achieve more rapid visual rehabilitation and experience fewer traumas.

The use of femtosecond lasers in cataract surgery has recently become popular. Femtosecond laser technology was initially used to create the flap in laser-assisted in situ keratomileusis (LASIK). [4] After being expanded to cataract surgery, femtosecond laser technology was used to perform lens fragmentation, anterior capsulotomy, and self-sealing corneal incisions. [5] Femtosecond laser-assisted cataract surgery (FLACS) offers numerous advantages over current surgical techniques. Studies have shown that use of FLACS leads to more accurate capsulorhexis than the manual procedure in CPS. [6,7] The quality of capsulorhexis has an effect on intraocular lens (IOL) position [8] and on the predictability in the IOL power calculation [5,9], thus affecting the visual and refractive outcomes. Previous studies have shown that the pre-treatment of cataracts with lasers using FLACS leads to a reduced IOL tilt and improved biometry predictability. [7,10,11] The corneal endothelium plays an important role in maintaining corneal transparency and normal thickness [12], and phacoemulsification time and energy are known to directly cause endothelial cell loss. [13–15] Many recent studies have found that FLACS helps to reduce effective phacoemulsification time (EPT) and the required phacoemulsification energy, thereby diminishing corneal endothelial injury. [16,17] Injury reduction of corneal endothelial cells contributes to shorten the recovery period and improve visual outcomes. [1,6] Based on the advantages of FLACS over CPS, some researchers have even predicted that the femtosecond laser will become the standard method of cataract extraction within ten years. [18]

The laser in FLACS is helpful for performing the self-sealing corneal incision, accurate capsulorhexis, and nuclear fragmentation. Notwithstanding the benefits of FLACS, many studies have compared the two techniques from several perspectives with varying results. Nevertheless, some ophthalmologists still doubt about the benefit from FLACS. [19]

There has only been one recently published meta-analysis [20] comparing the efficacy and safety of CPS and FLACS. The study included nine randomized controlled trials (RCTs) and concluded that FLACS resulted in significantly lower central corneal thickness (CCT) at one day follow-up, achieved a better corrected distant visual acuity (CDVA) at one week and six months postoperatively, and reduced phacoemulsification energy and EPT. However, because of the small sample size in this meta-analysis, the presence of bias and significant heterogeneity could not be ruled out. In addition, conducting a double blind RCT (i.e., in which patients and surgeons do not know which technique will be used before the operation) is sometimes unethical and difficult to carry out in clinical trials. A prospective randomized intraindividual cohort study [17] was also included in this RCT meta-analysis. To address the issue of small sample size and to provide more reliable and convincing evidence, a meta-analysis evaluating the differences in outcomes between FLACS and CPS was performed, with high-quality clinical cohort studies included.

## Materials and Methods

The meta-analysis was performed according to generally accepted methods. [21,22]

### Search strategy

PubMed, EMBASE, and the Cochrane Controlled Trials Register were searched for articles dated up to July 2015. A full-text search was conducted using the following terms: femtosecond OR femtolaser AND cataract. No restriction was placed on the language of the publication. The reference sections of the relevant reviews and original articles were also scanned for potential trials that may have been missed in the primary searches.

### Inclusion and exclusion criteria

Only reports of RCTs and comparative cohort studies comparing the outcomes of FLACS and CPS for age-related cataracts were included. Participants in the trials were patients with decreased visual acuity secondary to cataracts and no other eye disorders capable of compromising vision (e.g., amblyopia, glaucoma, diabetic retinopathy, or macular degeneration). At least one of the outcome measures in each included study was required.

### Screening process

Two independent reviewers (XY.C and KL.C) first conducted a preliminary review of the titles and abstracts; then, the full articles were analyzed to select the studies that met our predefined criteria. Disagreement between two reviewers was resolved through careful discussion—involving a third reviewer when necessary—until a consensus was reached.

### Quality assessment

Cochrane Collaboration’s tool for risk of bias [23] was used by two independent reviewers to evaluate the quality of the included RCTs. In short, the domains of sequence generation, allocation concealment, and selective outcome reporting were each addressed in the tool by a single entry for each study. The blinding of participants and personnel, the blinding of outcome assessment, and incomplete outcome data were also included in this tool. All of these parameters were graded as low risk of bias, high risk of bias, or unclear risk of bias, which indicated either a lack of information or uncertainty over the potential for bias.

The Newcastle-Ottawa Scale (NOS) [24] was used for quality assessment of the cohort study. This scale uses a total of nine stars: four in patient selection, two in comparability, and three in outcome assessment. A score ≥ 7 indicates good quality.

Two independent reviewers conducted this process, and discussion was used to resolve discrepancies.

### Data extraction

A standard form was used to extract the data, including the authors of each study, country and year of publication, study design, numbers, age and sex of patients, eye sample size, left-right eye proportion, follow-up duration and withdrawals. A second reviewer double-checked all data.

### Outcome measures

The measured outcomes included endothelial cell loss percentage, central corneal thickness, corrected distant visual acuity and uncorrected distant visual acuity, mean absolute error of refraction, surgically induced astigmatism, mean effective phacoemulsification time, phacoemulsification power, and circularity of the capsulorhexis.

For those studies that only reported absolute values for endothelial cell loss (ECL) count at the baseline and endpoint, endothelial cell loss percentage (ECL%) and the SD of the ECL% (SD_ECL%_) were calculated as follows:

$$\text{ECL}={\text{ECD}}_{\text{baseline}}-{\text{ECD}}_{\text{endpoint}};\;{\text{SD}}_{\text{ECL}}=\sqrt{{\text{SD}}_{\text{baseline}}^{2}{+\text{SD}}_{\text{endpoint}}^{2}-2\rho {\text{SD}}_{\text{baseline}}{\text{SD}}_{\text{endpoint}}}$$

where $\rho =\frac{\sigma_{\text{pre}}^{2}{-\sigma }_{\text{post}}^{2}-\sigma^{2}}{2\sigma_{\text{pre}}\sigma_{\text{post}}}$;

$$\text{ECL}\%=\frac{\text{ECL}\times 100\%}{{\text{ECD}}_{\text{baseline}}},\;{\text{SD}}_{\text{ECL}\%}=\frac{{\text{SD}}_{\text{ECL}}}{{\text{ECD}}_{\text{baseline}}}$$

For studies reporting visual outcomes not in the logMAR system but rather in the Snellen system: [25]

$${\text{BCVA}}_{\text{logMAR}}=-\text{log}\;{\text{BCVA}}_{\text{Snellen}},\;{\text{SD}}_{\text{logMAR}}=\text{log e}\times {\text{SD}}_{\text{Snellen}}(\text{when}\;{\text{BCVA}}_{\text{Snellen}}\approx 1)$$

### Statistical analysis

All statistical analyses were performed using RevMan software (version 5.3; Cochrane Collaboration, Oxford, United Kingdom). The weighted mean difference (WMD) with a 95% CI was calculated for the continuous outcomes. A p-value of less than 0.05 was considered statistically significant. Statistical heterogeneity was tested using the chi-square and I^2^. Fixed-effects models were used unless significant evidence of statistical heterogeneity or clinical diversity was found. However, for results showing significant heterogeneity (I^2^ > 50%), a random-effects meta-analysis was performed. Publication bias was measured using a Begg funnel plot. A sensitivity analysis was conducted to assess whether the results were affected by the excessive weight of a single study. The sensitivity analysis was performed by a one-study-removed analysis to assess how the results would change if a single study were omitted. [26]

## Results

### Literature search

**Fig 1:**
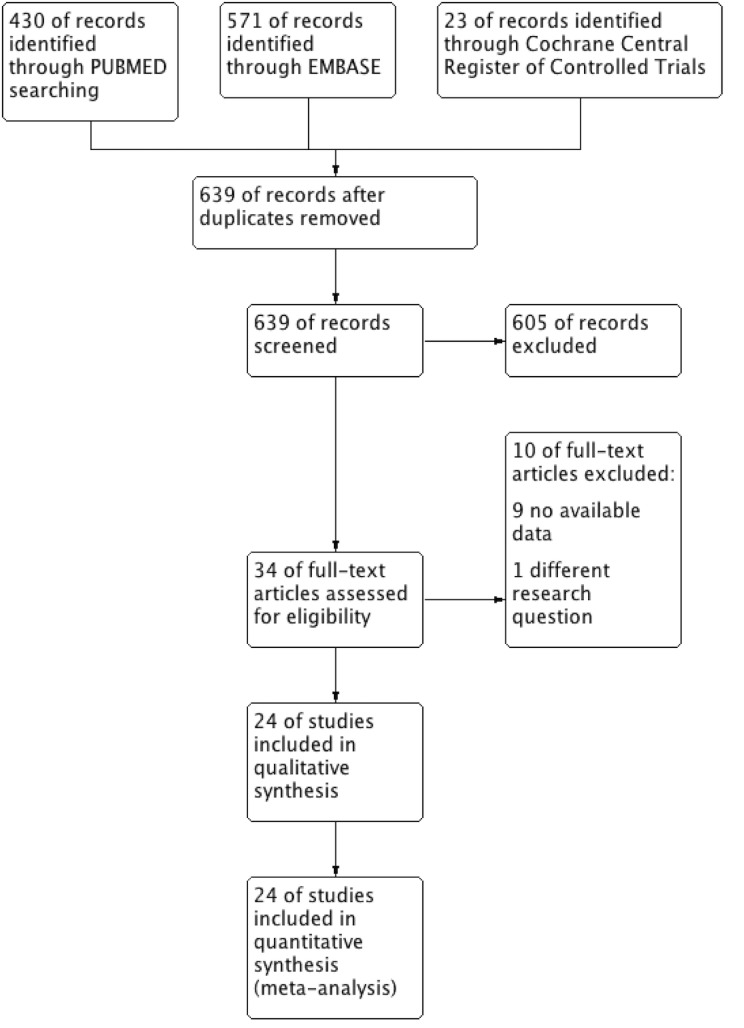
Flow diagram of the literature search in this meta-analysis.

The flowchart in Fig 1 shows the literature search process. After duplicates were removed, the titles and abstracts of 639 potentially relevant articles were scanned and 605 studies were excluded. Thirty-four full-text articles were then assessed for eligibility. Nine of them were excluded because the data were not relevant to our outcomes of interest, and one of them was excluded because it focused on another, different research question. Finally, 24 articles [9–11,16,17,27–45] meeting all of the predefined criteria were included in this meta-analysis.

### Characteristics of included studies

In the present meta-analysis, nine included studies were random clinical trials, and the other fifteen included studies were comparative cohort studies. Table 1 shows the characteristics of the 24 studies. The quality assessment of the RCTs is displayed in S1 Fig and that of the comparative cohort studies is shown in S1 Table. Overall, 4,903 eyes (2,861 assigned to the FLACS group and 2,072 assigned to the CPS group were analyzed. The mean age of the patients in these included studies ranged from 55 to 73 years. Four studies were completed in Australia, one in Singapore, one in India, and the remainder in Europe. Fifteen of these studies reported that approximately 44.2% of patients were male. The follow-up duration ranged from three weeks to six months.

**Table 1:** Characteristics of included studies. FLACS, femtosecond laser-assisted cataract surgery; CPS, conventional phacoemulsification surgery; RCT, randomized controlled trial; NA, not available.

Study ID	Country	Study design	Age		Sex (Male: Female)		No. of patients		No. of eyes		Follow-up
			FLACS	CPS	FLACS	CPS	FLACS	CPS	FLACS	CPS	
Abell 2013a	Australia	cohort	72.5±10.5		53:47	33:43	100	76	100	76	4 weeks
Takacs 2012	Hungary	RCT	65.81±12.42	66.93±0.99	10:28	15:23	38	38	38	38	1 month
Chee 2015	Singapore	cohort	64.5±9.86	65.5±9.39	NA	NA	NA	NA	794	420	6 weeks
Nagy 2011	Hungary	RCT	65±13	68±15	15:39	17:40	53	52	54	57	1 week
Filkorn 2012	Hungary	RCT	65.18±12.6	64.37±12.37	NA	NA	77	57	77	57	9 weeks
Conrad-Hengerer 2013	Germany	cohort	70.9	70.9	27:46	27:46	73	73	73	73	3 months
Conrad-Hengerer 2014a	Germany	cohort	72±10	73±10	NA	NA	400	400	400	400	NA
Ecsedy 2011	Hungary	cohort	58.85±15.27	66.85±11.77	8:12	5:15	20	20	20	20	1 month
Conrad-Hengerer 2012	Germany	cohort	70±11	72±8	30:27	28:24	57	52	57	52	4 weeks
Abell 2014	Australia	cohort	72.5±10.1		220:270		NA	NA	405	215	6 months
Reddy 2013	India	RCT	58.5±11.6	61.3±9.7	30:26	37:26	56	63	56	63	1 day
Krarup 2014	Denmark	cohort	NA	NA	NA	NA	47	47	47	47	3 months
Nagy 2014	Hungary	RCT	70.4±11.57	62.27±13.41	NA	NA	20	20	20	20	3 months
Mastropasqua 2014a	Italy	RCT	70.2±2.9	70.5±3.2	NA	NA	30	30	30	30	180 days
Schargus 2015	Germany	RCT	71.8	71.8	15:22	15:22	37	37	37	37	6 months
Conrad-Hengerer 2014b	Germany	RCT	71.3	71.3	46:58	46:58	104	104	104	104	6 months
Schultz 2015	Germany	cohort	70.96±9.89	69.94±10.48	25:25	23:27	50	50	50	50	3 days
Packer 2014	Germany	cohort	67.75±11.29	69.42±12.56	45:43	24:38	55	31	88	62	1 month
Nagy 2012	Hungary	cohort	55.17±17.25	62±14.27	7:5	5:8	12	13	12	13	2 months
Mastropasqua 2014b	Italy	RCT	69.3±3.4; 69.2±2.7	69.1±3.9	NA	NA	30+30	30	30+30	30	180 days
Lawless 2012	Australia	cohort	NA	NA	NA	NA	NA	NA	61	29	12 weeks
Abell 2013b	Australia	cohort	72.8±10.5	71.8±10.8	69:81	23:28	150	51	150	51	3 weeks
Daya 2014	UK	cohort	64.7±8.5	64.1±8.9	NA	NA	NA	NA	108	108	NA
Kranitz 2011	Hungary	cohort	63.78±13.97	71.69±11.34	5:15	6:14	20	20	20	20	1 year

### Primary outcomes

#### Endothelial cell loss percentage

**Fig 2:**
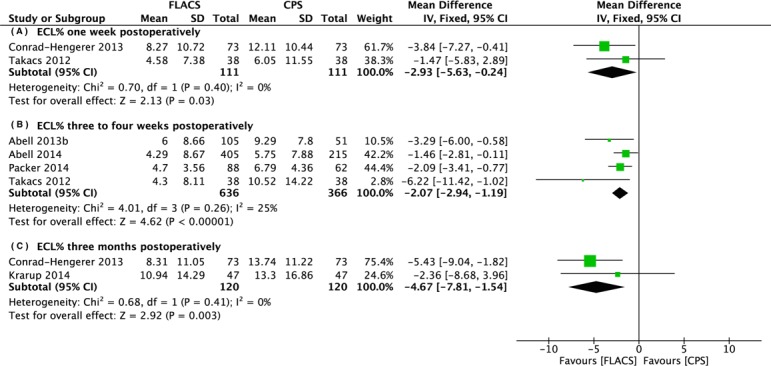
Forest plot comparison of ECL% after treatment with FLACS and CPS. (A) One week. (B) Three to four weeks. (C) Three months postoperatively.

There were seven studies reporting endothelial cell loss percentage at different time points. The mean ECL% in the FLACS group was significantly lower than that in the CPS group at one week (WMD: -2.93, 95% CI: -5.63 to -0.24, *P* = .03), approximately one month (WMD: -2.07, 95% CI: -2.94 to -1.19, *P* < .001), and three months (WMD: -4.67, 95% CI: -7.81 to -1.54, *P* = .003) postoperatively (Fig 2).

#### Central corneal thickness

**Fig 3:**
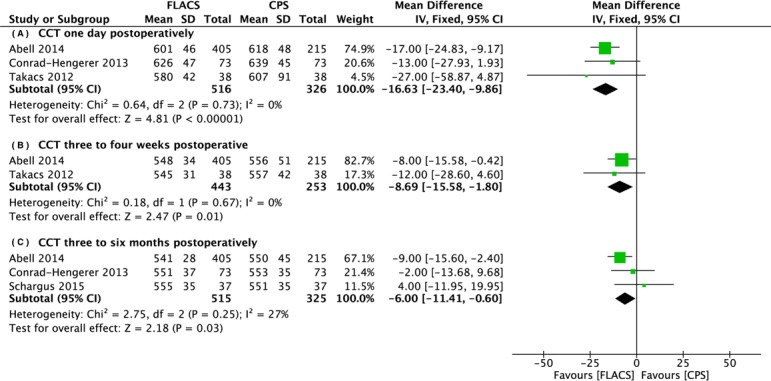
Forest plot comparison of CCT after treatment with FLACS and CPS. (A) One day. (B) Three to four weeks. (C) Three to six months postoperatively.

There were four studies reporting central corneal thickness at different time points. The thickness of the central cornea in the FLACS group was significantly lower than in the CPS group at one day (WMD: -16.63, 95% CI: -23.40 to -9.86, *P* < .001), approximately one month (WMD: -8.69, 95% CI: -15.58 to -1.80, *P* = .01), and three to six months (WMD: -6.00, 95% CI: -11.41 to -0.60, *P* = .03) postoperatively. (Fig 3)

#### Visual outcomes

**Fig 4:**
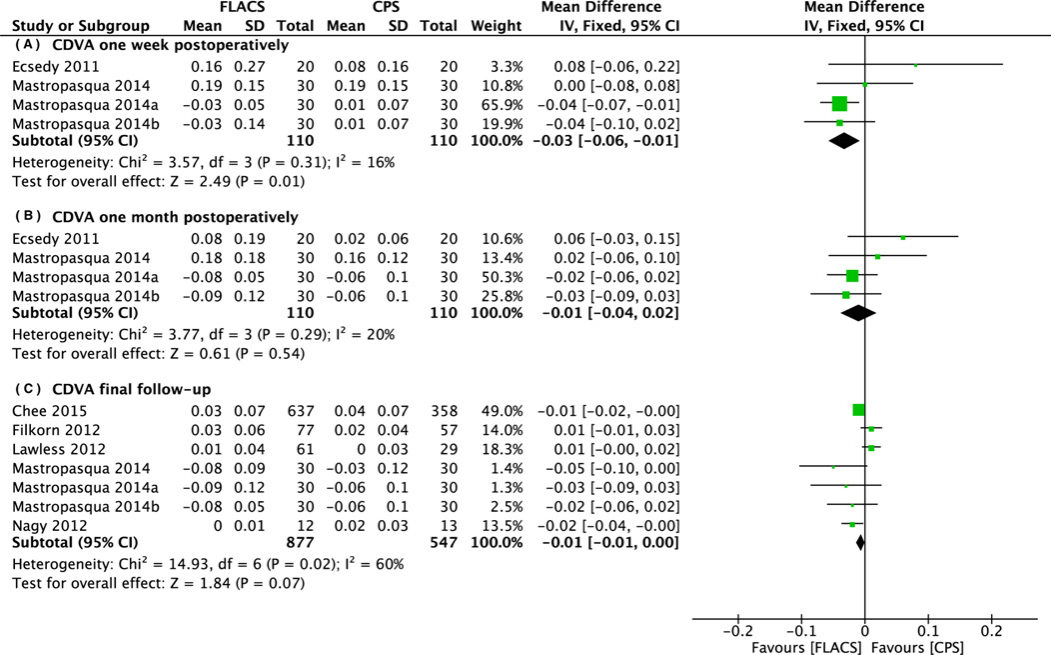
Forest plot comparison of CDVA after treatment with FLACS and CPS. (A) One week. (B) One month. (C) Final follow-up

**Fig 5:**
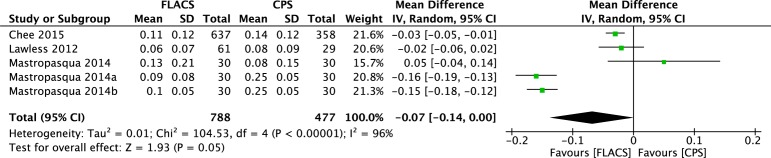
Forest plot comparison of UDVA at final follow-up after treatment with FLACS and CPS.

The corrected distant visual acuity was compared between the two groups at one week, one month, and at more than one month postoperatively (at the end of the follow-up period). The forest plots in Fig 4 shows that the CDVA in the FLACS group was significantly better than in the CPS group one week postoperatively (WMD: -0.03, 95% CI: -0.06 to -0.01, *P* = .01), and no significant differences of CDVA between the two groups one month postoperatively and at the end of the follow-up period (WMD: -0.01, 95% CI: -0.04 to 0.02, *P* = .54; WMD: -0.01, 95% CI: -0.01 to 0.00, 95% CI: -0.04 to 0.02, *P* = .07). However, the uncorrected distant visual acuity at the end of the follow-up period after FLACS appeared to be significantly better than after CPS (WMD: -0.07, 95% CI: -0.14 to 0.00, *P* = .05) based on a random-effects model (Fig 5).

#### Refractive outcomes

**Fig 6:**
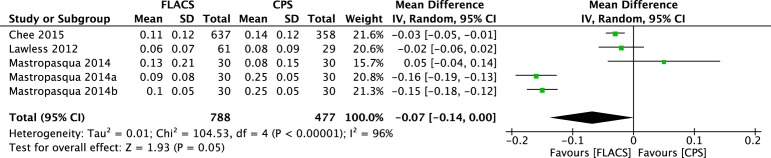
Forest plot comparison of MAE of spherical equivalent refraction after treatment with FLACS and CPS.

The mean absolute error of refraction is the difference between predicted and achieved postoperative spherical equivalent refraction, so mean absolute error (MAE) was used to analyze refractive outcomes. Six studies (including 1,696 eyes) compared the MAE after FLACS and CPS. The forest plot of the comparison in Fig 6 shows that the MAE of FLACS is significantly lower than that of CPS (WMD: -0.03, 95% CI: -0.06 to -0.01, *P* = .05).

### Secondary outcomes

#### Surgically induced astigmatism

Only two studies (including 100 eyes) compared surgically induced astigmatism (SIA) after FLACS and CPS. An analysis of these data showed no significant difference between the two groups (WMD: 0.05, 95% CI: -0.03 to 0.12, *P* = .26, I^2^ = 0%), as shown in S2 Fig.

#### Effective phacoemulsification time

Ten studies (including 1,174 eyes in the FLACS group and 1,027 eyes in the CPS group) compared effective phacoemulsification time during surgery. There was a significant difference between the two groups in favor of the FLACS group (WMD: -2.13, 95% CI: -2.60 to -1.66, *P* < .001, I^2^ > 50%), as shown in S3 Fig.

#### Phacoemulsification power

Two studies compared cumulative dissipated energy (CDE) and three studies compared mean phacoemulsification power (MP) between the FLACS and the CPS groups. Two subgroups (CDE and MP) were added to assess phacoemulsification power, as shown in S4 Fig. Seventy-seven eyes in the FLACS group and seventy-seven eyes in the CPS group showed that FLACS required significantly less cumulative dissipated energy (WMD: -2.23, 95% CI: -3.79 to -0.67, *P* = .005, I^2^ = 0%). Despite the high heterogeneity, studies reporting MP showed that the mean phacoemulsification power in the FLACS group was significantly lower than in the CPS group (WMD: -7.09, 95% CI: -7.64 to -6.55, *P* < .001, I^2^ > 50%). Therefore, the overall effect in phacoemulsification power favored FLACS (WMD: -6.57, 95% CI: -7.08 to -6.05, *P* < .001, I^2^ > 50%).

#### Circularity of capsulorhexis

Circularity is a parameter used for determining the regularity of capsulorhexis shape according to the following formula: circularity = 4π (area/perimeter^2^). Circularity values of 1.0 indicate a perfect circle. Four studies reported the circularity of capsulorhexis using the random effects model. The FLACS group had a significantly higher quality of circularity compared with the CPS group (WMD: 0.06, 95% CI: 0.03 to 0.09, P < .001, I^2^ > 50%), as shown in S5 Fig.

### Heterogeneity and publication bias

Some of the secondary outcomes displayed great heterogeneity. The heterogeneities of EPT, mean phacoemulsification power, and circularity were significant, and manually dropping eligible studies did not provide good results. No significant publication bias was demonstrated in the funnel plot.

## Discussion

The results of the present meta-analysis provide evidence that FLACS is safer, more accurate, causes fewer traumas, and achieves better visual outcomes than CPS. There were statistically significant differences in ECL%, CCT, CDVA at one day after surgery, UDVA at the end of the follow-up period, MAE, mean EPT, phacoemulsification power, and circularity of capsulorhexis between the FLACS and the CPS groups. However, no significant difference was found in surgically induced astigmatism between the two groups.

The CPS group had lost more endothelial cells than the FLACS group at one week, approximately one month, and three months postoperatively. Various factors can affect endothelial alterations including ultrasound time [46] and energy [47], irrigating solution [48], and the surgeon’s experience. Phacoemulsification time and energy are the most significant factors influencing endothelial cell damage. [13,14] In the present meta-analysis, both effective phacoemulsification time and phacoemulsification power favored the FLACS group. In addition, there was a direct relationship between endothelial cell loss and ultrasound power and time. [49] In contrast to the manual process of capsulorhexis and lens fragmentation in CPS, FLACS cataract pre-treatment reduces the amounts of potentially injurious ultrasound energy and time spent on lens emulsification. This means that the harmful effects of CPS on the endothelium are greater than those of FLACS. In addition, there is limited regenerative capacity of the corneal endothelium, a process that involves the self-repair of the endothelium through cell enlargement and migration. [12] That is, once the self-repair capacity of endothelium has been exceeded, long-term injury not only impacts the function of the endothelium but also its ability to withstand further injury. Therefore, when observing the different results at different time-points, it can be deduced that the reduction in EPT and phacoemulsification power not only contributes to reduced endothelial cell loss but also to the preservation of endothelial function and resistance to injury.

There was an increase in central corneal thickness in both groups, which reflects the development of central corneal edema after surgery. The corneal endothelium is responsible for maintaining corneal transparency and normal thickness. [12] Central corneal thickening always accompanies endothelial cell loss. When endothelial cell density significantly decreases, corneal edema may develop. [12] The central cornea in the CPS group was thicker than in the FLACS group at one day, one month, and three to six months postoperatively, suggesting that endothelial pump function was impaired, leading to corneal swelling. In addition, reduced corneal edema leads to faster visual recovery after IOL implantation. [17] This latter point is verified by our observation that the CDVA of patients in the FLACS group was much better at one week after surgery. However, a sensitivity analysis showed that if the final follow-up data from Abell’s 2014 study was excluded, the between-group differences became non-significant (WMD: 0.10, 95% CI: -9.33 to 9.52, *P* = .98, I^2^ = 0%). We infer that this is because the central corneas of patients in our CPS group were a little bit thicker than those of our FLACS group preoperatively, whereas in other articles, preoperative corneal thickness was nearly the same in both groups. Therefore, more experiments and additional data are required.

Visual rehabilitation is the most concerning problem for patients undergoing cataract surgery. FLACS leads to a better UDVA for patients at the final follow-up. CDVA is one of the best parameters for evaluating the quality and efficiency of a surgical technique. [46] Although there was no difference between the groups in the long-term comparison, the CDVA of the FLACS group was significantly better one week postoperatively, indicating that patients with FLACS can achieve rapid vision recovery, which is consistent with the central corneal thickness results. However, patients in both groups had excellent postoperative CDVA at the end of the follow-up period; this is why a significant difference was not observed between the CDVA values of the groups.

In the present meta-analysis, we observed that patients in the FLACS group obtained better circularity of capsulorhexis and experienced less mean absolute error than predicted. Previous studies have concluded that the type and shape of the capsulorhexis have a major effect on the IOL position. [9] The femtosecond laser-assisted capsulorhexis contributes to a more uniform shape, a more predictable size, and a more precise position. The improved capsulorhexis generally leads to a smaller variability in the actual IOL position compared to the precalculated one, which is expressed by a smaller MAE.

Besides the benefits and advantages of FLACS, financial issues should also be taken into consideration on the way of generalizing this new technology.[3] Some practitioners, especially well-skilled surgeons, still prefer CPS because they can also bring the patients good outcomes without the assistance of laser. And the extra dollars may affect their judgments on the choice of surgery procedure. However, as a clinician, better outcome consideration ranks above all else. Just like Australian patients’ free to extra FLACS costs, as the benefits of FLACS are proved, more and more insurance systems are thinking about covering these costs and then the cost considerations will mitigate. [18,19] As CPS gradually replaced extracapsular cataract extraction (ECCE), costs may decrease with the improvement and matureness of the technology. [19,50] And the wide adoption can amortize the capital costs. Therefore, FLACS will be more cost-effective and become a new option for surgeons and patients.

Inevitably, the present meta-analysis has several limitations. First, the limitations stem from the clinical trial itself. In some cases, it is not necessarily ethical for patients not to know which type of operation they will be undergoing. Cohort studies, which are not as reliable as RCTs, were therefore included in this meta-analysis. Second, these data were extracted from several trials, so it is difficult to unify the characteristics of patients, surgical conditions, surgeons, and data collection, all of which result in heterogeneity. Third, each of these studies solely focused on their particular fields of interest so that each outcome in this meta-analysis was derived from different studies.

## Conclusions

In conclusion, compared to conventional phacoemulsification surgery, femtosecond laser-assisted cataract surgery is safer and more effective in reducing endothelial cell loss and postoperative central corneal thickening and achieving better visual and refractive outcomes. However, there are no differences in SIA between the groups. Further studies with more outcomes and larger patient populations are needed to support our results.

## Supporting Information

S1 PRISMA Checklist PRISMA Checklist. (DOC)

S1 Table Quality assessment of cohort studies on the Newcastle-Ottawa Scale. (PDF)

S1 Fig Risk of bias graph. Review authors' judgments about each methodological quality item presented as percentages across included RCTs.
(TIF)

S2 Fig Forest plot comparison of SIA after treatment with FLACS and CPS. (TIF)

S3 Fig Forest plot comparison of EPT after treatment with FLACS and CPS. (TIF)

S4 Fig Forest plot comparison of phacoemulsification power after treatment with FLACS and CPS. (TIF)

S5 Fig Forest plot comparison of circularity of capsulorhexis after treatment with FLACS and CPS. (TIF)
