# Supplementary material for: Comparing the Curative Effects between Femtosecond Laser-Assisted Cataract Surgery and Conventional Phacoemulsification Surgery: A Meta-Analysis
Source: PLoS One. 2016 Mar 21;11(3):e0152088. doi: 10.1371/journal.pone.0152088 (PMC4801419; doi:10.1371/journal.pone.0152088)
Supplement: S1 Table — (PDF) [file pone.0152088.s002.pdf]

S1 Table. Quality assessment of cohort studies in Newcastle-Ottawa Scale.

		NOS scale			
Study ID	Country	Selection	Comparability	Outcome	Total score
Abell 2013	Australia	****	**	***	*********
Chee 2015	Singapore	****	**	**	********
Conrad-Hengerer 2013	Germany	****	**	***	*********
Conrad-Hengerer 2014a	Germany	****	*	***	********
Ecsedy 2011	Hungary	****	**	***	*********
Conrad-Hengerer 2012	Germany	****	*	***	********
Abell 2014	Australia	****	**	***	*********
Krarup 2014	Denmark	****	*	**	*******
Schultz 2015	Germany	****	**	**	********
Packer 2014	Germany	****	**	**	********
Nagy 2012	Hungary	****	**	***	*********
Lawless 2012	Australia	****	*	**	*******
Abell 2013b	Australia	****	**	**	********
Daya 2014	UK	****	*	**	*******
Kranitz 2011	Hungary	****	**	**	********

NOS, Newcastle-Ottawa Scale.
