# Supplementary figures and images for: Comparing the Curative Effects between Femtosecond Laser-Assisted Cataract Surgery and Conventional Phacoemulsification Surgery: A Meta-Analysis
Source: PLoS One. 2016 Mar 21;11(3):e0152088. doi: 10.1371/journal.pone.0152088 (PMC4801419; doi:10.1371/journal.pone.0152088)

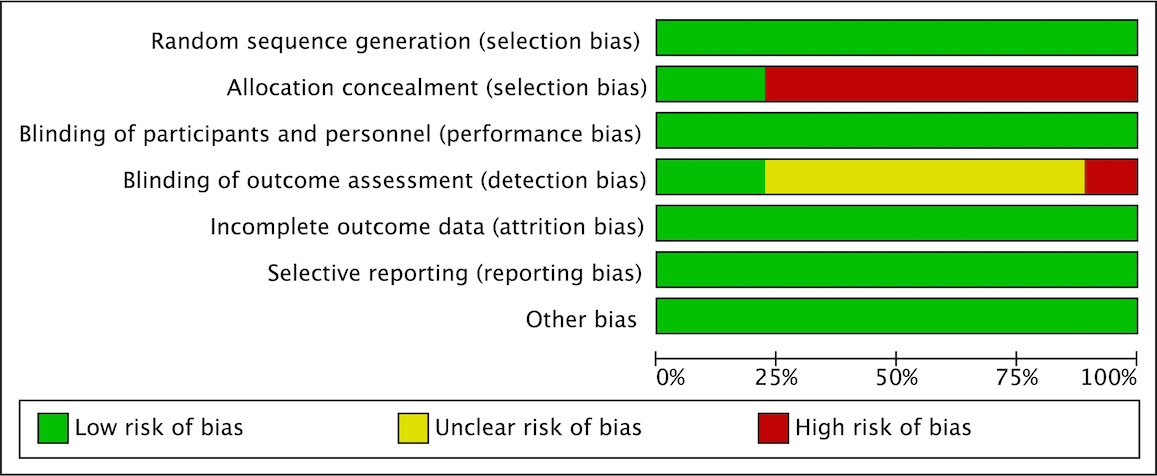

Supplement: S1 Fig — Review authors' judgments about each methodological quality item presented as percentages across included RCTs. (TIF) [file pone.0152088.s003.tif]

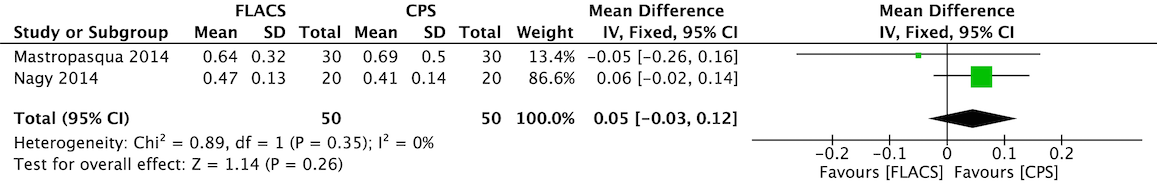

Supplement: S2 Fig — (TIF) [file pone.0152088.s004.tif]

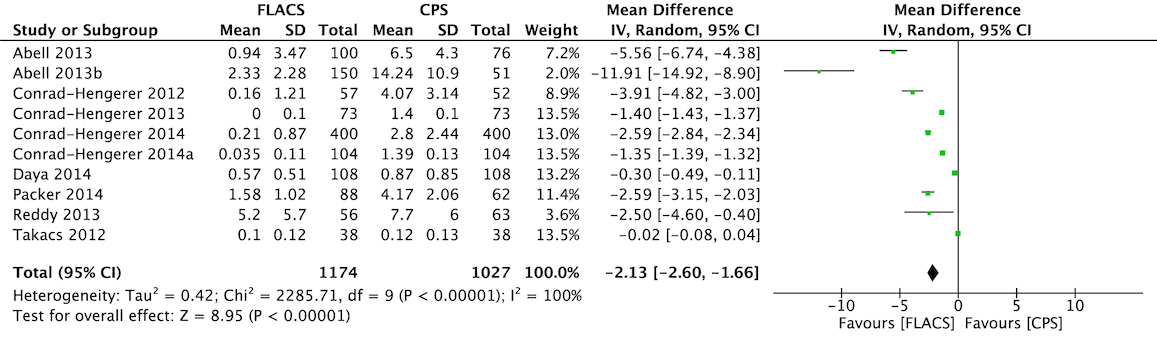

Supplement: S3 Fig — (TIF) [file pone.0152088.s005.tif]

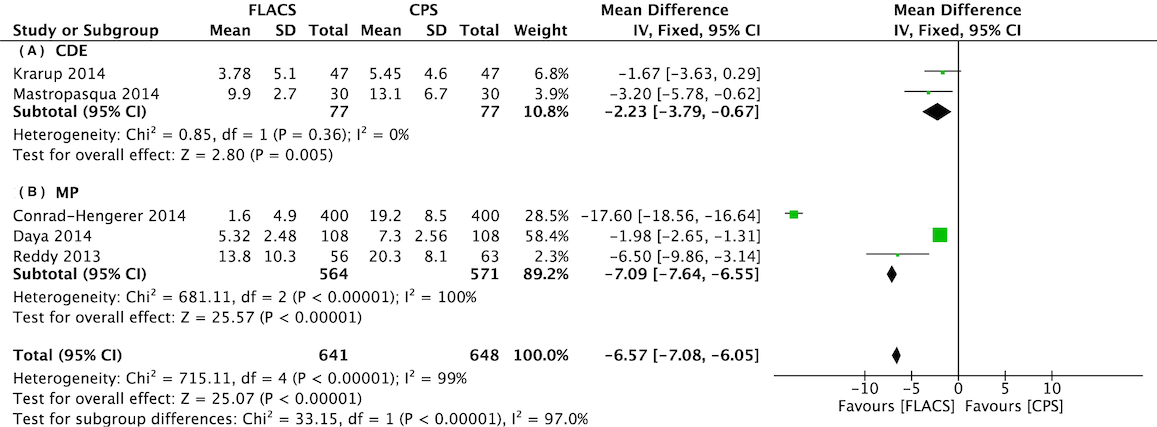

Supplement: S4 Fig — (TIF) [file pone.0152088.s006.tif]

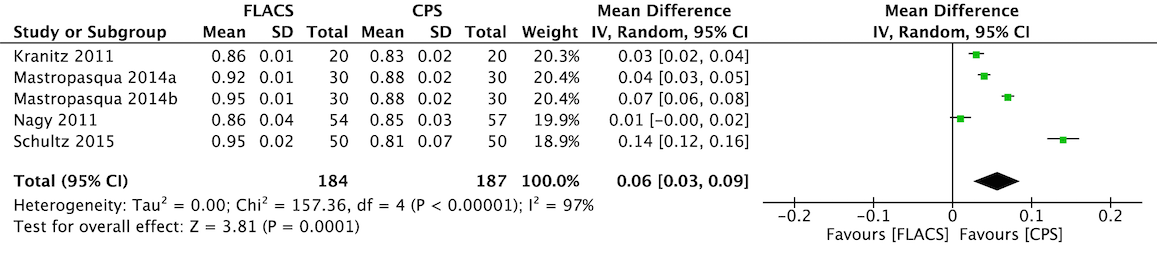

Supplement: S5 Fig — (TIF) [file pone.0152088.s007.tif]
